# Dental Caries

**Published:** 1891-10

**Authors:** 


### Dental Caries.

The following prescription has been suggested:

R. Acid tannic.................................lxxv grains.

Tinct. iodinii ...............................

Tinct. myrrhæ, aa.........................j fluid drachm.

Potassii iodidi..............................xv grains.

Aquæ rosæ...................................j fluid ounce. M.

Add a teaspoonful to a small glass of water and use daily as a mouth wash.—*Pharm. Record*, March 5, 1891.

